# Systemic Stress Biomarkers and Patient-Reported Outcomes Following Laparoscopic IPOM+ and Open Rives–Stoppa Repair for Ventral Incisional Hernia: A Prospective Non-Randomized Comparative Study

**DOI:** 10.3390/jcm15187198

**Published:** 2026-09-16

**Authors:** Bojan Jovanović, Vanja Pecić, Ljubiša Rančić, Marija Dinić, Ksenija Marković, Nataša Milić, Nenad Lalović, Nebojša Ignjatović

**Affiliations:** 1 Center for Minimally Invasive Surgery, University Clinical Center Niš, 18000 Niš, Serbiamarija9696dinic@gmail.com (M.D.); 2 Faculty of Medicine, University of Niš, 18000 Niš, Serbia; 3 Institute for Medical Statistics and Informatics, Faculty of Medicine, University of Belgrade, 11000 Belgrade, Serbia; 4 University Hospital Foča, 73300 Foča, Bosnia and Herzegovina; 5 Faculty of Medicine Foča, University of East Sarajevo, 73300 Foča, Bosnia and Herzegovina; 6 Center for Digestive Surgery, University Clinical Center Niš, 18000 Niš, Serbia

**Keywords:** incisional hernia, laparoscopic IPOM+, Open Rives–Stoppa repair, EuraHS-QoL, biomarkers

## Abstract

**Introduction:** Ventral incisional hernia repair remains a significant surgical challenge, with ongoing debate regarding the optimal approach. While laparoscopic intraperitoneal onlay mesh (IPOM+) and open Rives–Stoppa techniques are widely utilized, a comprehensive understanding integrating perioperative clinical outcomes, patient-reported quality of life, and the systemic stress response is lacking. This study aimed to compare these two surgical techniques across these multifaceted domains. **Methods:** This prospective non-randomized comparative study enrolled 96 adult patients (*n* = 48 per group) undergoing elective ventral incisional hernia repair via either laparoscopic IPOM+ or open Rives–Stoppa techniques. Perioperative clinical outcomes (operative time, hospital stay, bowel function, pain, analgesic use, complications, self-reported return to daily activities) and EuraHS-QoL scores were assessed. A comprehensive panel of endocrine (cortisol, ACTH, prolactin), inflammatory (IL-6, CRP, presepsin, procalcitonin), and coagulation (D-dimer) biomarkers were measured preoperatively and on the first postoperative day. **Results:** Laparoscopic IPOM+ repair was associated with more favorable postoperative recovery compared to open repair, characterized by reduced pain, shorter hospital stay, faster return to daily activities and better patient-reported quality of life (all *p* < 0.001). No significant time-by-group interactions were observed for endocrine or coagulation markers, indicating comparable perioperative trajectories (*p* > 0.05), while the inflammatory response (IL-6, C-reactive protein, presepsin, and procalcitonin) differed between the two approaches. Significant time-by-group interactions were found for IL-6 (*p* = 0.005), C-reactive protein (*p* = 0.015), presepsin (*p* = 0.040) and procalcitonin (*p* = 0.006). These associations remained after adjustment for baseline age, BMI, employment status and ASA classification. However, following Bonferroni’s adjustment for multiple comparisons, the observed associations between the biomarkers and the outcome did not remain statistically significant. **Conclusions:** Laparoscopic IPOM+ was associated with more favorable postoperative recovery and lower one-month EuraHS-QoL scores compared to the open Rives–Stoppa repair. Although some biomarkers suggested a possible difference in the postoperative inflammatory response between the two groups, further studies are needed to determine whether these findings reflect true biological differences between the surgical techniques.

## 1. Introduction

Incisional hernia remains one of the most common long-term complications of abdominal surgery, with reported incidence rates varying considerably according to patient characteristics, surgical procedure, and the duration of follow-up [1]. Beyond the anatomical defect, incisional hernias substantially impair patients’ quality of life because of chronic pain, impaired physical function, altered body image, and limitations in daily activities while also imposing a considerable socioeconomic burden [1,2]. Tension-free mesh reinforcement is currently considered the standard of care for ventral incisional hernia repair, as recommended by the European Hernia Society and the Americas Hernia Society, because it significantly reduces long-term recurrence rates [3,4]. Open retromuscular repair in the Rives–Stoppa (RS) plane remains one of the most widely accepted techniques for many large and complex defects because it provides durable reconstruction with mesh placement in a well-vascularized extraperitoneal plane [5]. In contrast, laparoscopic ventral hernia repair has consistently been associated with lower wound morbidity, fewer surgical-site infections, and shorter hospital stay compared with conventional open repair [6]. However, laparoscopic intraperitoneal onlay mesh (IPOM+) repair is not without limitations. The placement of an intraperitoneal mesh has raised concerns regarding mesh–bowel adhesions, erosion, fistula formation, and chronic pain, although the introduction of modern composite meshes and meticulous surgical techniques has substantially reduced these risks. Consequently, careful patient selection and appropriate surgical expertise remain essential when considering the IPOM+ approach. Increasing emphasis has been placed on Patient-Reported Outcome Measures (PROMs), recognizing that successful ventral hernia repair should be evaluated not only by recurrence and postoperative complications but also by patients’ quality of life and functional recovery [7]. Validated instruments, such as the European Hernia Society Quality-of-Life (EuraHS-QoL) questionnaire, are routinely used to assess postoperative pain, activity restriction, and aesthetic satisfaction following ventral hernia repair [8]. Beyond patient-reported outcomes, the biological response to surgical trauma has emerged as another important determinant of postoperative recovery [9]. Tissue injury initiates a complex neuroendocrine, inflammatory, and metabolic cascade characterized by activation of the innate immune system and release of pro-inflammatory cytokines, particularly interleukin-6 (IL-6), which subsequently stimulates hepatic synthesis of C-reactive protein (CRP) [9,10]. The magnitude of the postoperative inflammatory response has been associated with adverse postoperative outcomes, including delayed recovery, postoperative complications, and prolonged hospitalization following abdominal surgery [10,11]. Several patient- and procedure-related factors have also been identified as contributors to postoperative complications after ventral hernia repair [12]. Circulating biomarkers provide complementary insight into the physiological stress response to surgery. Cortisol reflects activation of the hypothalamic–pituitary–adrenal axis, whereas IL-6 and CRP represent key indicators of postoperative inflammatory activity [9,13]. In addition, D-dimer reflects activation of coagulation and fibrinolysis, while procalcitonin and presepsin have emerged as promising biomarkers of systemic inflammatory activation in the perioperative setting. Together, these biomarkers provide an objective assessment of the endocrine, inflammatory, and coagulative consequences of surgical trauma [9].

Recent evidence suggests that minimally invasive abdominal surgery is associated with a reduced systemic inflammatory response compared with conventional open procedures, primarily owing to less tissue injury and lower operative stress [10,14]. Nevertheless, despite the growing body of evidence supporting this biological advantage, data specifically evaluating stress biomarkers in ventral incisional hernia repair remain limited [10]. Although recent studies have increasingly focused on clinical outcomes and patient-reported quality of life after ventral incisional hernia repair, only a limited number have simultaneously evaluated perioperative clinical outcomes, patient-reported quality of life, and objective endocrine, inflammatory, and coagulation-related biomarkers of surgical stress within the same prospective patient cohort [10,13,15]. Consequently, the relationship between surgical approach, biological stress response, and postoperative recovery in ventral incisional hernia repair has not yet been fully elucidated. Therefore, the aim of the present study was to compare laparoscopic IPOM+ and open Rives–Stoppa repair in patients with ventral incisional hernia by evaluating perioperative clinical outcomes, postoperative quality of life, and perioperative changes in endocrine, inflammatory, and coagulation-related stress biomarkers.

## 2. Materials and Methods

### 2.1. Study Design and Setting

This prospective non-randomized comparative clinical and biochemical study was conducted at the Center for Minimally Invasive Surgery, University Clinical Center Niš, Serbia. Consecutive adult patients requiring elective surgical repair for ventral incisional hernias between June 2024 and June 2025 were prospectively enrolled. The study protocol received formal approval from the Institutional Ethics Committee of the University Clinical Center Niš (Approval No. 11846/7). Written informed consent was obtained from all individual participants prior to study enrollment and any data collection. The study cohort comprised 96 patients: 48 consecutive patients undergoing laparoscopic IPOM+ repair and 48 consecutive patients undergoing open Rives–Stoppa repair were prospectively enrolled. The operative approach was determined in routine clinical practice and was not assigned by the investigators. Laparoscopic IPOM+ repair was performed by one surgeon experienced in this technique, whereas open Rives–Stoppa repair was performed by other surgeons. Patients were assigned to a surgeon according to the routine outpatient appointment schedule, and the final operative approach was selected after clinical evaluation. No randomization or allocation concealment was used.

### 2.2. Patient Selection

Eligible participants included adult patients diagnosed with a primary midline ventral incisional hernia who were scheduled for elective mesh repair. Inclusion criteria were defined as follows: age ≥ 18 years; primary midline ventral incisional hernia classified as W2 (defect width 4–10 cm) according to the European Hernia Society (EHS) classification; and eligibility for elective laparoscopic IPOM+ or open Rives–Stoppa repair. None of the patients had undergone previous hernia repair. All procedures were performed by experienced abdominal wall surgeons with more than 15 years of operative experience to ensure technical standardization. All patients underwent a one-year postoperative clinical follow-up, including physical examination to assess postoperative complications and hernia recurrence.

### 2.3. Surgical Techniques

#### 2.3.1. Laparoscopic IPOM+ Repair

Laparoscopic IPOM+ repair was performed under general endotracheal anesthesia using one 10 mm and two 5 mm trocars, with pneumoperitoneum maintained at approximately 12 mmHg. After adhesiolysis and complete reduction in the hernia contents, the fascial defect was measured after reducing the pneumoperitoneum pressure to 8 mmHg and closed intracorporeally with a continuous non-absorbable size 0 polypropylene suture. A 20 × 15 cm dual-component composite mesh was placed intraperitoneally and fixed to the anterior abdominal wall with absorbable tacks in a double-crown configuration. After verification of hemostasis and mesh position, the pneumoperitoneum was released and the trocar sites were closed.

#### 2.3.2. Open Rives-Stoppa Repair

Open Rives–Stoppa repair was performed under general endotracheal anesthesia through an incision along the previous surgical scar. After reduction in the hernia sac, the retromuscular space was developed bilaterally and the posterior fascial layer was closed with a continuous non-absorbable size 0 polypropylene suture. A 20 × 15 cm polypropylene mesh was placed in the retromuscular plane and fixed with interrupted slowly absorbable 2-0 sutures, followed by closure of the anterior fascial layer above the mesh. A drain was routinely placed in the retromuscular and/or suprafascial space before layered wound closure.

### 2.4. Anesthetic and Analgesic Management

All procedures were performed under general balanced endotracheal anesthesia using the same basic protocol in both groups. Anesthesia was induced intravenously with propofol and fentanyl and maintained with sevoflurane in an oxygen–air mixture or, when clinically indicated, total intravenous anesthesia. Standard intraoperative monitoring and lung-protective mechanical ventilation were used in both groups, with ventilatory parameters adjusted during pneumoperitoneum in the laparoscopic IPOM+ group. Analgesia consisted of intravenous ketoprofen, paracetamol, and metamizole administered according to pain intensity and individual contraindications. The first dose of analgesia was administered after the patient returned to the surgical ward, while subsequent doses were given as needed according to pain intensity. Analgesic therapy consisted of metamizole, paracetamol, ketoprofen, or a combination of metamizole and paracetamol. Analgesic doses were expressed according to the amount of active drug per administration: metamizole 2.5 g per ampoule, paracetamol 1 g per 100 mL infusion, and ketoprofen 100 mg per ampoule. Total postoperative analgesic consumption was recorded over the entire postoperative hospitalization, until discharge.

### 2.5. Clinical and Perioperative Data Collection

Baseline demographic and clinical characteristics, including age, sex, body mass index (BMI), and comorbidities (hypertension, diabetes mellitus, cardiovascular disease, and other comorbid conditions), were prospectively recorded. Perioperative variables included operative time, length of postoperative hospital stay, time to restoration of bowel movement (duration of postoperative ileus), intraoperative complications (bleeding and bowel injury), postoperative pain assessed using a 0–10 point Visual Analogue Scale (VAS) after 24 h, analgesic consumption and time to return to daily activities, and postoperative complications, including wound seroma and mesh infection, were prospectively recorded. Time to usual daily activities was defined as the number of postoperative days until employed patients first resumed light occupational duties or, for retired and unemployed patients, until they resumed their usual light daily activities. This outcome was obtained by patient self-report during postoperative follow-up, was not independently verified, and was considered exploratory.

### 2.6. Biomarkers of Surgical Stress and Inflammation

To objectively assess the physiological response to surgical trauma, peripheral venous blood samples were collected preoperatively and on the first postoperative day. Both samples were obtained routinely at 07:00 after an overnight fast. The preoperative sample was collected before surgery and before administration of anesthetic or intravenous analgesic medications, whereas the postoperative sample was collected before administration of chronic therapy or intravenous medications, including analgesics. No patient had clinical evidence of infection at the time of blood sampling. The biochemical panel included neuroendocrine stress markers (cortisol, adrenocorticotropic hormone [ACTH], and prolactin [PRL]), inflammatory biomarkers (interleukin-6 [IL-6], C-reactive protein [CRP], procalcitonin [PCT], and white blood cell count [WBC]), presepsin, the coagulation marker D-dimer, and blood glucose as a metabolic stress marker. All samples were processed and analyzed at the Institute of Biochemistry, University Clinical Center Niš, according to standardized laboratory protocols.

### 2.7. Patient-Reported Outcomes (EuraHS-QoL)

Health-related quality of life was assessed one month postoperatively using the standardized European Hernia Society Quality of Life (EuraHS-QoL) questionnaire. The EuraHS-QoL is a disease-specific questionnaire comprising nine items that evaluate pain, restriction of activities, and cosmetic discomfort, with a total score ranging from 0 (best quality of life) to 90 (worst quality of life. The Serbian-language version of the EuraHS-QoL questionnaire used in this study was previously applied by Antić et al. [16]. Clinical follow-up also included physical examinations to assess for hernia recurrence and late postoperative complications.

### 2.8. Sample Size Calculation

The target sample size was powered based on the incidence of surgical site infections (primary outcome) reported by Liang et al., with rates of 7.5% following laparoscopic repair and 34.5% following open ventral incisional hernia repair [17]. Assuming a two-sided significance level (α) of 0.05 and a statistical power (1-β) of 90%, the minimum required sample size was 48 patients per group, yielding a total study population of 96 patients. This study was not specifically powered to detect between-group differences in other clinical outcomes, EuraHS-QoL scores or biomarker concentrations (secondary outcomes).

### 2.9. Statistical Analysis

Numeric variables were expressed as mean ± standard deviation (SD), standard error (SE) or 95% confidence interval (95% CI). Categorical variables were presented as absolute frequencies and percentages. Intergroup comparisons of numeric variables were performed using the independent-samples Student’s *t*-test. Categorical variables were compared using the Pearson chi-square test or Fisher’s exact test, as appropriate. The internal consistency of the EuraHS-QoL questionnaire was assessed using Cronbach’s alpha coefficient. No more than 5% of the data were missing for any of the variables, and no data imputation was performed. Changes in perioperative stress biomarkers between the preoperative and postoperative measurements were analyzed using repeated-measure analysis of variance (ANOVA). To account for the baseline difference in age, BMI, employment status and ASA score between the study groups, repeated-measure analysis of covariance (ANCOVA) was additionally performed. To account for multiple comparisons, Bonferroni’s correction was applied by dividing the conventional significance level of 0.05 by the total number of statistical analyses yielding *p*-values. As 42 statistical comparisons were performed, the adjusted significance threshold was set at 0.0012 (0.05/42). Statistical analyses were performed using IBM SPSS Statistics for Windows, version 29.0 (IBM Corp., Armonk, NY, USA).

## 3. Results

This study included 96 patients, with 48 patients in each surgical group. Baseline demographic and clinical characteristics according to the surgical technique are presented in Table 1. Men comprised approximately half of the study population, accounting for 50.0% of patients in the IPOM+ group and 54.2% in the RS group. The mean age was 52.52 ± 11.73 years in the IPOM+ group and 58.56 ± 12.82 years in the RS group, while the mean BMI was 25.62 ± 2.95 kg/m^2^ and 26.75 ± 2.65 kg/m^2^, respectively. Smoking was reported in 43.8% of patients in the IPOM+ group and 47.9% in the RS group, whereas comorbidities were present in 62.5% and 77.1% of patients, respectively. Employment was reported in 64.6% in the IPOM+ group and 47.9% of patients in the RS group. Regarding ASA physical status, ASA II was the most frequent category in both groups (52.1% in the IPOM+ group and 58.3% in the RS group), followed by ASA I (37.5% vs. 20.8%) and ASA III (10.4% vs. 18.8%). Overall, baseline characteristics were well balanced between the groups. Cohen’s d values ranged from 0.08 to 0.49 in absolute magnitude, indicating very small to small standardized differences between the IPOM+ and RS groups. The largest imbalance was observed for age (d = −0.492) and BMI (d = −0.403), although these still represented small effect sizes according to Cohen’s conventional thresholds. The overall differences in employment status and ASA distribution were also small (h = 0.337 and Cramér’s V = 0.177, respectively), while differences in sex and smoking status were very small (h = −0.083 and h = −0.083). Hernia size was comparable between the groups, with only a very small standardized difference (Cohen’s d = 0.149).

Operative time and postoperative outcomes differed significantly between the two surgical groups (Table 2). The IPOM+ group had a significantly shorter operative time compared with the Rives–Stoppa (RS) group (55.00 ± 12.38 vs. 75.10 ± 13.70 min; difference: −20.104 min, 95% CI: −25.396 to −14.812; *p* < 0.001; d = −1.540). Postoperative hospital stay was also significantly shorter in the IPOM+ group (1.83 ± 0.83 vs. 3.58 ± 1.67 days; difference: −1.750 days, 95% CI: −2.283 to −1.217; *p* < 0.001; d = −1.332). Similarly, bowel function recovery occurred earlier following IPOM+ repair (1.21 ± 0.68 vs. 1.90 ± 0.81 days; difference: −0.688 days, 95% CI: −0.990 to −0.385; *p* < 0.001; d = −0.921). Postoperative pain scores were significantly lower in the IPOM+ group compared with the RS group (3.88 ± 0.79 vs. 6.63 ± 1.02; difference: −2.750, 95% CI: −3.120 to −2.380; *p* < 0.001), with the largest effect size observed for this outcome (d = −3.009). Overall, all observed differences remained statistically significant after adjustment for age, BMI, employment status, and ASA classification, with large effect sizes across all evaluated outcomes (Supplemental Table S1). The results remained unchanged after applying the Bonferroni correction for multiple testing.

Intraoperative and postoperative complications according to the surgical technique are presented in Table 3. Overall, complications occurred less frequently in patients undergoing IPOM+ than in those treated with the RS technique. Intraoperative complications were recorded in 4.2% of patients in the IPOM+ group compared with 6.3% in the RS group, while postoperative complications occurred in 8.3% and 20.8% of patients, respectively. Despite these lower complication rates following laparoscopic repair, the standardized effect sizes were small (0.093 for intraoperative and 0.356 for postoperative complications). The results remained unchanged after adjustment for age, BMI, employment status, and ASA classification and applying the Bonferroni correction for multiple testing (Supplemental Table S2).

The incidence of surgical site infection was negligible in both groups, indicating a similarly low risk of postoperative wound infection regardless of the surgical technique. Most postoperative complications were minor (Clavien–Dindo grade I–II). In addition, only one patient developed recurrent incisional hernia during follow-up, and this occurred in the RS group (Table 4). No patient experienced more than one complication; therefore, the number of patients with complications corresponded to the total number of complications.

EuraHS-QoL total score and its domains according to the surgical technique are presented in Table 5. The EuraHS-QoL instrument demonstrated good internal consistency, with a Cronbach’s alpha coefficient of 0.874. Patients undergoing IPOM+ repair had lower scores across all EuraHS-QoL domains compared with patients undergoing RS repair. The cosmetic score was lower in the IPOM+ group (7.04 ± 2.67 vs. 13.50 ± 3.24, *p* < 0.001), with a very large standardized effect size (Cohen’s d = −2.176). Similarly, activity restriction score was lower after IPOM+ repair (12.67 ± 3.94 vs. 23.25 ± 4.65, *p* < 0.001; Cohen’s d = −2.456), as was the pain score (8.58 ± 3.24 vs. 16.52 ± 2.97, *p* < 0.001; Cohen’s d = −2.554). The total EuraHS-QoL score was also lower in the IPOM+ group (28.29 ± 8.55 vs. 53.27 ± 9.78, *p* < 0.001; Cohen’s d = −2.719), indicating substantially better postoperative quality of life. In addition, patients undergoing IPOM+ resumed light work or usual daily activities earlier than those undergoing RS repair (5.17 ± 0.95 vs. 12.21 ± 3.00 days, *p* < 0.001). This difference was accompanied by a very large standardized effect size (Cohen’s d = −3.161) (Supplemental Table S3). After adjustment for potential confounding factors, including age, body mass index (BMI), and the presence of comorbidities, and applying the Bonferroni correction for multiple testing, the observed differences between IPOM+ and RS repair remained unchanged, confirming the robustness of the findings (Supplemental Tables S3 and S4).

**Figure 1:**
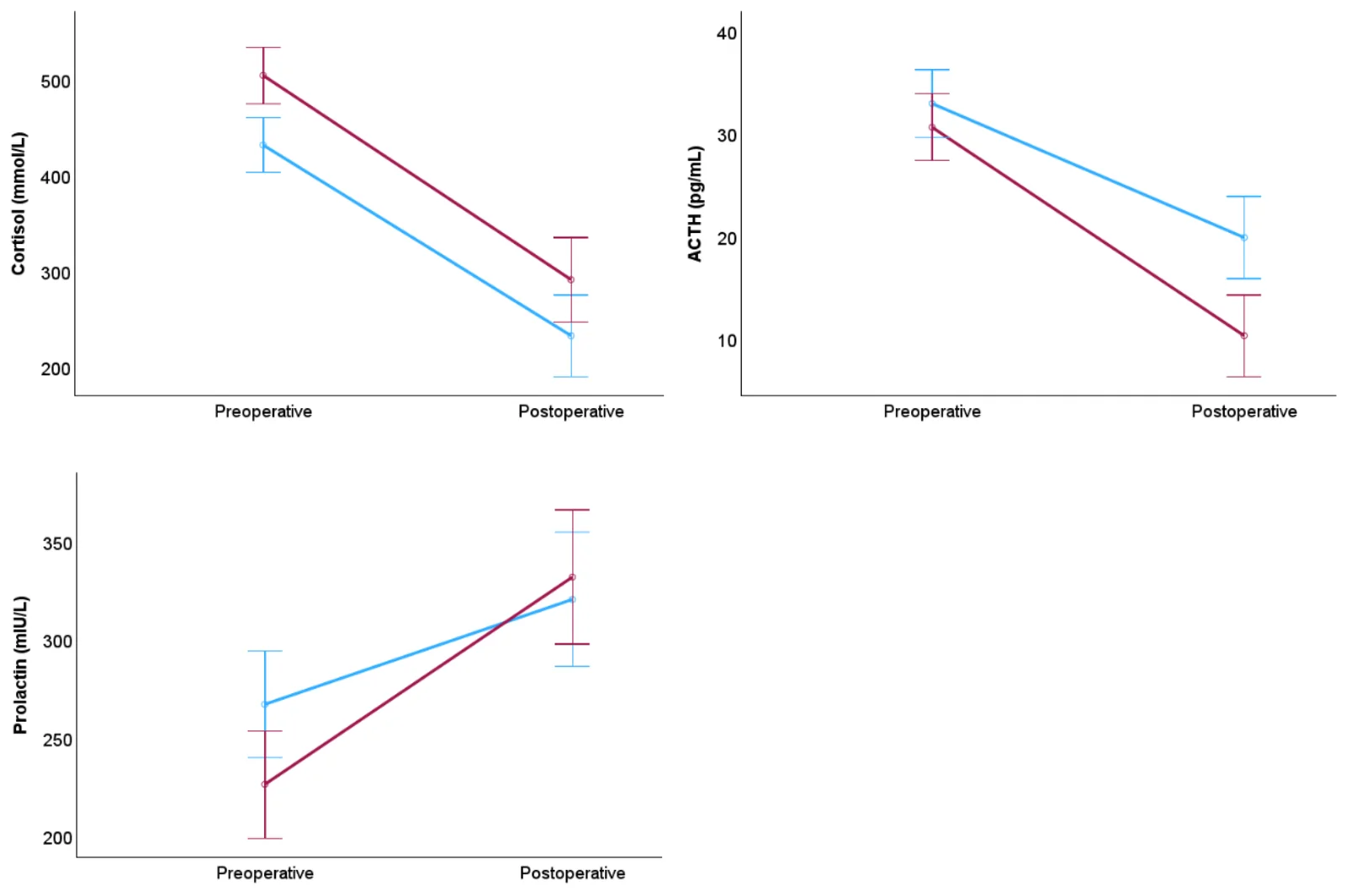
Preoperative and postoperative endocrine biomarker levels according to the surgical technique (values are adjusted for age, BMI, comorbidities and ASA status); Blue line: Laparoscopic IPOM+, Red line: Open Rives–Stoppa (RS) repair; error bars: ±1SE.

**Figure 2:**
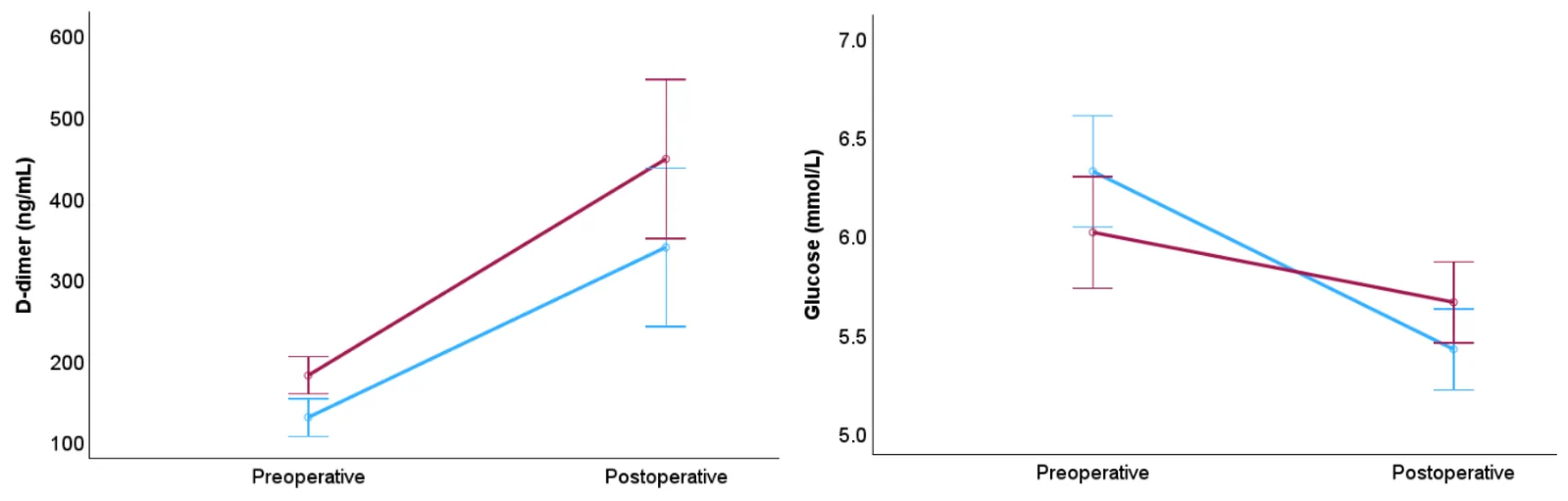
Preoperative and postoperative coagulation (D-dimer) and metabolic (Glucose) biomarker levels according to the surgical technique (values are adjusted for age, BMI, comorbidities and ASA status); Blue line: Laparoscopic IPOM+, Red line: Open Rives–Stoppa (RS) repair; error bars: ±1SE.

**Figure 3:**
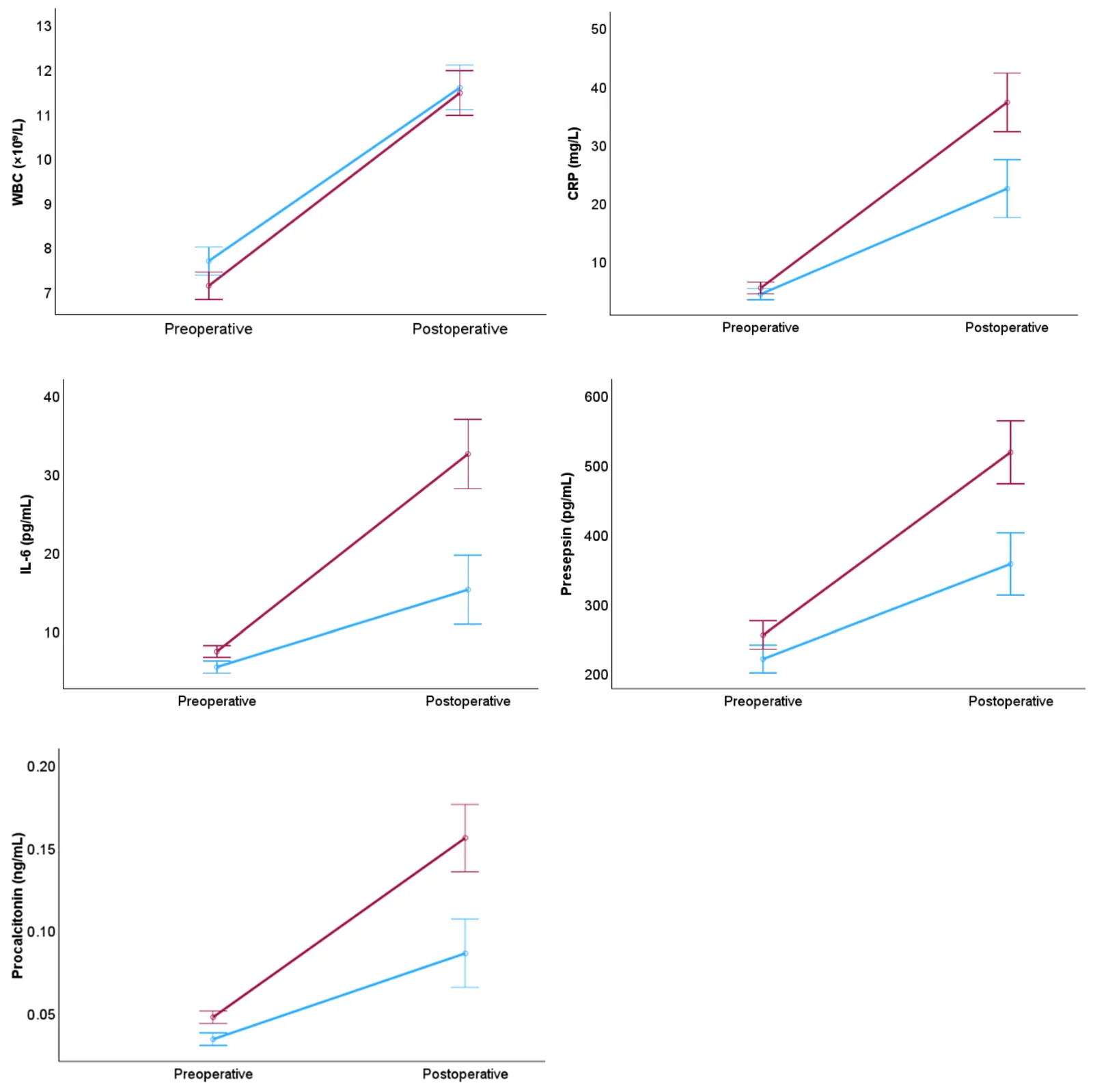
Preoperative and postoperative inflammatory biomarker levels according to the surgical technique (values are adjusted for age, BMI, comorbidities and ASA status); Blue line: Laparoscopic IPOM+, Red line: Open Rives–Stoppa (RS) repair; error bars: ±1SE.

A repeated-measure ANOVA was performed to evaluate the effects of time, surgical approach, and their interaction on biomarker concentrations (Table 6). Significant time × surgical approach interactions with moderate effect sizes were observed for IL-6 (*p* = 0.005, partial ηp^2^ = 0.082), CRP (*p* = 0.015, partial ηp^2^ = 0.062), presepsin (*p* = 0.040, partial ηp^2^ = 0.044), and procalcitonin (*p* = 0.006, partial ηp^2^ = 0.079), indicating that the magnitude of postoperative change differed significantly between the IPOM+ and RS groups, with greater increases observed in the RS group. No significant time × surgical approach interactions were observed for cortisol (*p* = 0.805, partial ηp^2^ = 0.001), ACTH (*p* = 0.413, partial ηp^2^ = 0.007), prolactin (*p* = 0.103, partial ηp^2^ = 0.028), WBC (*p* = 0.117, partial ηp^2^ = 0.026), D-dimer (*p* = 0.421, partial ηp^2^ = 0.007), or glucose (*p* = 0.383, partial ηp^2^ = 0.008). After the adjustment (Supplemental Table S5), significant time × surgical approach interactions remained for IL-6 (*p* = 0.016, partial ηp^2^ = 0.063), CRP (*p* = 0.043, partial ηp^2^ = 0.045), presepsin (*p* = 0.041, partial ηp^2^ = 0.046) and procalcitonin (*p* = 0.050, partial ηp^2^ = 0.042), indicating that the magnitude of postoperative change in these biomarkers differed significantly between the surgical groups, with greater increases observed in the RS group. No significant adjusted time × surgical approach interactions were observed for cortisol (*p* = 0.849, partial ηp^2^ < 0.001), ACTH (*p* = 0.333, partial ηp^2^ = 0.011), prolactin (*p* = 0.219, partial ηp^2^ = 0.017), WBC (*p* = 0.454, partial ηp^2^ = 0.006), D-dimer (*p* = 0.649, partial ηp^2^ = 0.003), or glucose (*p* = 0.177, partial ηp^2^ = 0.020). Preoperative and postoperative biomarker levels adjusted for age, BMI and comorbidities are presented in Figure 1, Figure 2 and Figure 3 according to surgical technique. However, after applying the Bonferroni correction for multiple testing, none of the evaluated biomarkers reached statistical significance.

## 4. Discussion

The present prospective nonrandomized comparative study comprehensively evaluated perioperative clinical outcomes, patient-reported quality of life, and the systemic stress response in patients undergoing elective ventral incisional hernia repair using either laparoscopic IPOM+ or open Rives–Stoppa repair. Our findings demonstrate that laparoscopic IPOM+ repair was associated with more favorable postoperative recovery, characterized by reduced pain, shorter hospital stay, lower one-month EuraHS-QoL scores and faster return to daily activities. Although both surgical techniques elicited comparable endocrine and coagulation responses, the inflammatory response differed between the two approaches. Specifically, IL-6, C-reactive protein, presepsin, and procalcitonin showed perioperative changes according to the surgical technique, whereas cortisol, ACTH, prolactin, white blood cell count, D-dimer, and blood glucose exhibited parallel trajectories. Importantly, these associations remained after adjustment for baseline age, BMI and presence of comorbidities, although residual confounding by other measured or unmeasured factors cannot be excluded. However, following Bonferroni’s adjustment for multiple comparisons, none of the observed time × surgical approach interaction effects remained statistically significant.

Surgical trauma inevitably activates a complex neuroendocrine response, influencing the hypothalamic–pituitary–adrenal (HPA) axis and leading to metabolic and immunological changes [9]. Activation of the HPA axis is influenced not only by tissue trauma but also by general anesthesia, pneumoperitoneum, carbon dioxide insufflation, and increased intra-abdominal pressure. Despite the well-established clinical advantages of laparoscopic ventral hernia repair over open surgery, including reduced postoperative pain and faster recovery, endocrine responses may remain comparable because of the physiological effects of pneumoperitoneum and anesthesia [18,19]. These perioperative factors contribute to the overall physiological stress response, potentially attenuating differences in endocrine biomarker responses between minimally invasive and open procedures [9,20]. Consequently, endocrine biomarkers appear to reflect the overall physiological burden of surgery rather than solely the degree of local tissue dissection. In contrast, inflammatory biomarkers are more closely associated with the magnitude of tissue trauma and activation of the innate immune response, making them particularly useful indicators for evaluating the biological impact of different surgical techniques [21,22,23]. The greater postoperative inflammatory response observed after open Rives–Stoppa reconstruction is biologically expected. This technique necessitates extensive retromuscular dissection, significant abdominal wall mobilization, and considerable tissue manipulation, all of which promote cytokine release and activate the acute phase response [5,24]. In contrast, the laparoscopic IPOM+ technique involves less extensive abdominal wall dissection than open Rives–Stoppa repair, which may contribute to reduced local tissue trauma and a less pronounced systemic inflammatory response. Similar observations have been reported in systematic reviews, demonstrating that minimally invasive abdominal surgery is associated with a reduced postoperative inflammatory response compared with conventional open procedures [14].

The postoperative behavior of presepsin and procalcitonin was also consistent with the observed differences in inflammatory response between the groups. Although both biomarkers were originally introduced for the diagnosis of sepsis, growing evidence indicates that they also reflect the intensity of sterile postoperative inflammation and activation of the innate immune response following major surgical trauma [21,22,24]. In the present study, these were associated with the surgical approach; however, these findings do not establish that the laparoscopic technique directly caused a less pronounced inflammatory response. Collectively, these findings suggest that attenuation of the postoperative inflammatory response may represent a potential contributing factor to the more favorable clinical recovery observed in the laparoscopic group; however, this hypothesis requires confirmation through formal mediation analysis. Excessive postoperative inflammation has consistently been associated with increased postoperative pain, delayed functional recovery, prolonged hospitalization, and a slower return to normal daily activities [11]. The biomarker profile observed in our study may provide a plausible biological context for the more favorable perioperative recovery observed after laparoscopic IPOM+ repair. This interpretation is consistent with comparative evidence reporting improved short-term postoperative recovery following minimally invasive ventral hernia repair [25,26,27].

Changes in coagulation biomarkers represent another important component of the physiological response to surgical trauma. In the present study, D-dimer levels increased significantly after surgery in both groups, reflecting the expected activation of coagulation and fibrinolysis following tissue injury [9,10]. However, no significant differences in perioperative D-dimer trajectories were observed between laparoscopic IPOM+ and open Rives–Stoppa repair. This finding is biologically plausible because the two techniques involve distinct but partially counterbalancing physiological responses. Open surgery is associated with greater tissue dissection and endothelial injury, whereas laparoscopic repair requires carbon dioxide pneumoperitoneum and increased intra-abdominal pressure, both of which have been shown to induce transient venous stasis and activation of the coagulation cascade despite reduced tissue trauma [9,19]. Consequently, D-dimer appears to reflect the overall physiological response to surgery rather than differences between surgical approaches. Despite comparable perioperative coagulation activation, patients in the laparoscopic group experienced faster postoperative recovery, including earlier mobilization, shorter hospital stay, and earlier resumption of daily activities, indicating an association between the minimally invasive approach and more favorable short-term clinical outcomes.

The biological differences observed between the two surgical techniques were accompanied by clinically meaningful improvements in postoperative recovery. Patients undergoing laparoscopic IPOM+ repair had shorter operative time, earlier restoration of bowel function, lower postoperative pain scores, reduced analgesic requirements, and shorter hospital stay than those treated with the open Rives–Stoppa technique. These findings are consistent with contemporary evidence demonstrating that minimally invasive ventral hernia repair is associated with superior short-term postoperative outcomes, including reduced postoperative pain, shorter hospital stay, faster recovery, and comparable surgical efficacy when compared with open repair [27,28,29]. In light of the substantial clinical and socioeconomic burden of incisional hernias, these short-term advantages support further evaluation and refinement of minimally invasive abdominal wall reconstruction, with the aim of reducing surgical trauma while preserving effective and durable repair [29,30]. Although laparoscopic IPOM+ offers well-established short-term clinical advantages, the intraperitoneal placement of mesh remains a subject of ongoing discussion because of the potential risk of mesh-related complications, including adhesion formation, bowel erosion, enterocutaneous fistula, bowel obstruction, and chronic postoperative pain. However, advances in composite mesh technology with anti-adhesive barriers, together with routine fascial defect closure in the IPOM+ technique, may have contributed to an improved safety profile of intraperitoneal mesh placement, and contemporary evidence suggests that these complications are uncommon when appropriate patient selection and surgical technique are applied [3,15,28].

Beyond conventional perioperative outcomes, patient-reported outcome measures have become essential indicators of treatment success following abdominal wall reconstruction [7]. Accordingly, postoperative quality of life was assessed using the disease-specific EuraHS-QoL questionnaire developed by the European Hernia Society [8]. The importance of incorporating disease-specific quality-of-life assessment into the evaluation of ventral and incisional hernia repair has been further emphasized by recent prospective studies demonstrating that EuraHS-QoL provides a sensitive measure of postoperative recovery and patient-perceived treatment success [30]. These observations reinforce the concept that abdominal wall reconstruction should be evaluated not solely through traditional surgical metrics, but also through patient-centered outcomes that better reflect the overall impact of surgery on daily functioning and well-being.

Patients who underwent laparoscopic IPOM+ repair reported better quality of life at one month and returned to daily activities approximately one week earlier than those treated with the open Rives–Stoppa technique. The EuraHS-QoL questionnaire is particularly valuable in this setting because it specifically evaluates hernia-related pain, activity restriction, and cosmetic discomfort, thereby providing a more comprehensive assessment of treatment effectiveness than conventional perioperative measures alone [30,31]. These findings are consistent with recent prospective studies and systematic reviews demonstrating that minimally invasive ventral hernia repair is associated with lower postoperative pain, faster functional recovery, more favorable postoperative quality of life, and earlier return to normal daily activities [30,31,32]. Furthermore, a recent systematic review and meta-analysis demonstrated that laparoscopic ventral hernia repair reduced the time to return to work by nearly six days compared with open repair, with all included studies consistently favoring the minimally invasive approach [33]. Similar findings were also reported in comparative studies evaluating laparoscopic IPOM/IPOM+ and open mesh repair, which documented significantly earlier return to work following laparoscopic surgery [34].

### Limitations

This study has several limitations. First, it was conducted at a single tertiary referral center, which may limit the generalizability of the findings. In addition, its non-randomized design, and the operative approach was determined in routine clinical practice by the treating surgeon. Because laparoscopic IPOM+ repair was performed by one surgeon and open Rives–Stoppa repair by other surgeons, selection bias, confounding by indication, and surgeon-related effects cannot be excluded. The postoperative assessment of stress biomarkers was based on a single postoperative day 1 measurement, which does not allow for evaluation of their complete temporal kinetics or peak concentrations. Therefore, the absence of significant differences in biomarkers at this timepoint should not be interpreted as evidence of equivalent overall biological responses. In addition, presepsin and procalcitonin are not specific markers of sterile inflammation, and an infection-related contribution cannot be completely excluded. Although maximum defect width of hernia was recorded, the second defect dimension, calculated area, detailed morphology, and loss of domain were not available. Preoperative EuraHS-QoL scores were not obtained, and the time to resumption of light work or usual daily activities was self-reported and should be considered exploratory. Finally, follow-up focused mainly on early postoperative recovery; long-term outcomes such as chronic pain, and long-term quality of life were not evaluated.

## 5. Conclusions

Laparoscopic IPOM+ was associated with more favorable patient recovery and quality of life compared with the open Rives–Stoppa repair. Although some biomarkers suggested a possible difference in the postoperative inflammatory response between the two groups, further studies are needed to determine whether these findings reflect true biological differences between the surgical techniques. This study underscores the importance of combining objective biomarkers with patient-reported outcomes for a more comprehensive evaluation of surgical techniques and postoperative recovery in future abdominal wall reconstruction research.

## Figures and Tables

**Table 1:** Baseline demographic and clinical characteristics according to the surgical technique.

Variable	IPOM+ (*n* = 48)	RS (*n* = 48)	Difference (95% CI)	Effect Size
Male sex, *n* (*p*)	24 (0.50)	26 (0.54)	−0.042 (−0.246 to 0.163)	−0.083 (very small)
Age (yrs), mean ± SD	52.52 ± 11.73	58.56 ± 12.82	−6.042 (−11.020 to −1.063)	−0.492 (small)
BMI (kg/m^2^), mean ± SD	25.62 ± 2.95	26.75 ± 2.65	−1.129 (−2.265 to 0.006)	−0.403 (small)
Smoking, *n* (*p*)	21 (0.44)	23 (0.48)	−0.042 (−0.235 to 0.155)	−0.083 (very small)
Comorbidities, *n* (*p*)	30 (0.62)	37 (0.77)	−0.146 (−0.332 to 0.040)	−0.318 (small)
Employment, *n* (*p*)	31 (0.65)	23 (0.48)	0.167 (−0.034 to 0.367)	0.337 (small)
ASA, *n* (%)				
I	18 (37.5)	11 (22.9)	14.6 (−3.6 to 32.7)	0.177 (small)
II	25 (52.1)	28 (58.3)	−6.3 (−26.1 to 13.6)	
III	5 (10.4)	9 (18.8)	−8.3 (−22.4 to 5.7)	
Hernia size (cm), mean ± SD	6.9 ± 1.9	6.7 ± 1.9	0.279 (−0.479 to 1.038)	0.149 (very small)

ASA, American Society of Anesthesiologists Physical Status Classification; IPOM+, laparoscopic intraperitoneal onlay mesh technique; RS, open Rives–Stoppa technique; p, proportion; CI, confidence interval. Effect size: Cohen’s d for continuous variables, Cohen’s h for dichotomous categorical variables, and Cramér’s V for ASA classification.

**Table 2:** Operative time and postoperative outcomes according to the surgical technique.

Variable	IPOM+ (*n* = 48) Mean ± SD	RS (*n* = 48) Mean ± SD	Difference (95% CI)	*p*	Effect Size
Operative time (min)	55.00 ± 12.38	75.10 ± 13.70	−20.104 (−25.396 to −14.812)	**<0.001**	−1.540 (large)
Postoperative hospital stay (days)	1.83 ± 0.83	3.58 ± 1.67	−1.750 (−2.283 to −1.217)	**<0.001**	−1.332 (large)
Bowel function recovery (days)	1.21 ± 0.68	1.90 ± 0.81	−0.688 (−0.990 to −0.385)	**<0.001**	−0.921 (large)
Postoperative pain score (VAS, 0–10)	3.88 ± 0.79	6.63 ± 1.02	−2.750 (−3.120 to −2.380)	**<0.001**	−3.009 (large)

Data are presented as mean ± standard deviation or mean difference (95% confidence interval for mean). Effect size corresponds to Cohen’s d, calculated using the pooled standard deviation. Negative values indicate lower values in the IPOM+ group relative to the RS group. Bold values indicate statistical significance after Bonferroni’s correction.

**Table 3:** Intraoperative and postoperative complications according to surgical technique.

Complications	IPOM+ (*n* = 48) *n* (%)	RS (*n* = 48) *n* (%)	Difference (95% CI)	*p*	Effect Size
Intraoperative	2 (0.042)	3 (0.063)	−0.021 (−0.112 to 0.070)	0.646	−0.093 (very small)
Postoperative	4 (0.083)	10 (0.21)	−0.125 (−0.267 to 0.017)	0.083	−0.356 (small)

Effect size: Cohen h for dichotomous categorical variables.

**Table 4:** Type and Clavien Dindo classification of postoperative complications according to surgical technique.

Variable	IPOM+ (*n* = 48)	RS (*n* = 48)
Surgical site infection	0	1
Seroma	4	9
Recurrent incisional hernia	0	1
Clavien-Dindo classification		
Grade 1	3	3
Grade 2	1	6
Grade 3	0	2

**Table 5:** Postoperative EuraHS-QoL scores according to the surgical technique.

EuraHS-QoL Score	IPOM+ (*n* = 48) Mean ± SD	RS (*n* = 48) Mean ± SD	Difference (95% CI)	*p*	Effect Size
Total	28.29 ± 8.55	53.27 ± 9.78	−24.979 (−28.703 to −21.255)	**<0.001**	−2.719 (large)
Pain	8.58 ± 3.24	16.52 ± 2.97	−7.937 (−9.179 to −6.678)	**<0.001**	−2.554 (large)
Restriction of activities	12.67 ± 3.94	23.25 ± 4.65	−10.583 (−12.330 to −8.837)	**<0.001**	−2.456 (large)
Cosmetic	7.04 ± 2.67	13.50 ± 3.24	−6.458 (−7.661 to −5.256)	**<0.001**	−2.176 (large)

Data are presented as mean ± standard deviation or mean (95% confidence interval for mean). Effect size corresponds to Cohen d. Bold values indicate statistical significance after Bonferroni’s correction.

**Table 6:** Preoperative and postoperative biomarker levels according to surgical technique and time × group interaction effects.

Biomarker		IPOM+ (*n* = 48)	RS (*n* = 48)	*p*	Effect Size
Cortisol (nmol/L)	preoperative	425.57 ± 24.16	509.99 ± 31.53	0.805	0.001 small
	postoperative	228.09 ± 36.44	295.40 ± 46.50		
	change (95%CI)	197.5 (111.5 to 283.4)	214.6 (104.0 to 325.2)		
ACTH (pg/mL)	preoperative	33.34 ± 3.47	30.22 ± 2.94	0.413	0.007 small
	postoperative	19.54 ± 5.25	10.52 ± 1.70		
	change (95%CI)	13.80 (0.86 to 26.74)	19.70 (13.12 to 26.28)		
Prolactin (mIU/L)	preoperative	272.9 ± 35.80	219.82 ± 12.80	0.103	0.028 small
	postoperative	320.0 ± 37.70	332.22 ± 28.47		
	change (95%CI)	−47.0 (−103.9 to 9.8)	−112.4 (−168.4 to −56.4)		
IL-6 (pg/mL)	preoperative	5.06 ± 0.41	7.58 ± 1.05	0.005	0.082 moderate
	postoperative	13.94 ± 2.33	33.73 ± 5.72		
	change (95%CI)	−8.88 (−13.65 to −4.11)	−23.15 (−37.16 to −15.14)		
WBC (×10^9^/L)	preoperative	7.71 ± 0.34	7.10 ± 0.25	0.117	0.026 small
	postoperative	11.36 ± 0.51	11.67 ± 0.49		
	change (95%CI)	−3.65 (−4.35 to −2.96)	−4.58 (−5.52 to −3.63)		
D-dimer (ng/mL)	preoperative	122.17 ± 16.84	187.51 ± 26.75	0.421	0.007 small
	postoperative	312.76 ± 38.45	472.89 ± 127.97		
	change (95%CI)	−190.6 (−249.0 to −132.2)	−285.4 (−514.0 to −56.8)		
Glucose (mmol/L)	preoperative	6.14 ± 0.32	6.20 ± 0.24	0.383	0.008 small
	postoperative	5.34 ± 0.21	5.74 ± 0.20		
	change (95%CI)	0.80 (0.16 to 1.43)	0.46 (0.03 to 0.89)		
CRP (mg/L)	preoperative	4.12 ± 0.83	5.47 ± 1.12	0.015	0.062 moderate
	postoperative	21.26 ± 3.89	38.29 ± 5.74		
	change (95%CI)	−17.14 (−24.76 to −9.53)	−32.82 (−43.08 to −22.56)		
Presepsin (pg/mL)	preoperative	225.47 ± 18.38	248.76 ± 21.84	0.040	0.044 small
	postoperative	364.81 ± 32.57	509.47 ± 52.80		
	change (95%CI)	−139.3 (−201.4 to −77.3)	−260.7 (−361.1 to −160.3)		
Procalcitonin (ng/mL)	preoperative	0.034 ± 0.003	0.047 ± 0.005	0.006	0.079 small
	postoperative	0.074 ± 0.012	0.167 ± 0.026		
	change (95%CI)	−0.04 (−0.06 to −0.02)	−0.12 (−0.17 to −0.07)		

Data are presented as mean ± standard error or mean (95% confidence interval for mean). Change was calculated as the preoperative value minus the postoperative value; therefore, negative values indicate a postoperative increase. Effect size corresponds to Partial ηp^2^. The *p* values and partial eta squared (ηp^2^) values refer to the time × surgical approach interaction obtained using repeated-measure ANOVA. Bold values indicate statistical significance after Bonferroni’s correction.

## Data Availability

Aggregate data are presented in this manuscript. De-identified individual participant data are available from the corresponding author upon reasonable request, subject to approval by the Institutional Ethics Committee and applicable data protection regulations.

## Supplementary Materials

The following supporting information can be downloaded at: https://www.mdpi.com/article/10.3390/jcm15187198/s1, Table S1. Adjusted operative time and postoperative outcomes according to the surgical technique; Table S2. Adjusted intraoperative and postoperative complications according to surgical technique; Table S3. Adjusted postoperative EuraHS-QoL scores according to the surgical technique; Table S4. Nonadjusted and adjusted time to resumption of daily activities (days) according to the surgical technique; Table S5. Adjusted preoperative and postoperative biomarker levels according to surgical approach and time × group interaction effects.

## References

[1] Omar I., Zaimis T., Townsend A., Ismaiel M., Wilson J., Magee C. (2023). Incisional Hernia: A Surgical Complication or Medical Disease?. Cureus.

[2] Hill S., Bullock J., Sanders D.L. (2023). Quality of Life With a Hernia-A Novel Patient Led Study. J. Abdom. Wall Surg..

[3] Hatewar A., Mahakalkar C., Kshirsagar S., Ram Sohan P., Dixit S., Bikkumalla S. (2024). From Meshes to Minimally Invasive Techniques: A Comprehensive Review of Modern Hernia Repair Approaches. Cureus.

[4] Henriksen N.A., Montgomery A., Kaufmann R., Berrevoet F., East B., Fischer J., Hope W., Klassen D., Lorenz R., Renard Y. (2020). Guidelines for treatment of umbilical and epigastric hernias from the European Hernia Society and Americas Hernia Society. Br. J. Surg..

[5] Rhemtulla I.A., Fischer J.P. (2018). Retromuscular Sublay Technique for Ventral Hernia Repair. Semin. Plast. Surg..

[6] Ajii D.J., Akpagher S.F., Amaechi O.V., Matthew O., Udochukwu B.O. (2026). Comparative outcomes of laparoscopic versus open ventral hernia repair: A systematic review and meta-analysis. Hernia.

[7] Mills J.M.Z., Luscombe G.M., Hugh T.J. (2024). Long-term patient-reported outcome measures (PROMs) after primary ventral or small midline incisional hernia repair. ANZ J. Surg..

[8] Muysoms F., Campanelli G., Champault G.G., DeBeaux A.C., Dietz U.A., Jeekel J., Klinge U., Köckerling F., Mandala V., Montgomery A. (2012). EuraHS: The development of an international online platform for registration and outcome measurement of ventral abdominal wall hernia repair. Hernia.

[9] Acharya K., Rout D.K., Kapadia N.A., Caroicar Y.S., Karunakaran N.B., Patel P., Thaker H. (2025). Surgical Stress Response: A Physiological Review of the Endocrine, Immune, and Metabolic Changes. Cureus.

[10] Kokotovic D., Burcharth J., Helgstrand F., Gögenur I. (2017). Systemic inflammatory response after hernia repair: A systematic review. Langenbeck’s Arch. Surg..

[11] Kovoor J.G., Bacchi S., Nathin K., Aujayeb N., Lu A., Mishra N.C., Tyagi D., Stretton B., Gupta A.K., Hugh T.J. (2025). Postoperative inflammatory responses and association with in-hospital mortality after general surgery. ANZ J. Surg..

[12] Assakran B.S., Al-Harbi A.M., Abdulrahman Albadrani H., Al-Dohaiman R.S. (2024). Risk Factors for Postoperative Complications in Hernia Repair. Cureus.

[13] Janet J., Derbal S., Durand Fontanier S., Bouvier S., Christou N., Fabre A., Fredon F., Rivaille T., Valleix D., Mathonnet M. (2021). C-reactive protein is a predictive factor for complications after incisional hernia repair using a biological mesh. Sci. Rep..

[14] Keating T., Drumm C., Kennedy N., Cullinane C., Condon E., Fitzgerald M., Peirce C., Coffey J.C., Fleming C.A. (2026). Systemic inflammatory response after robotic versus laparoscopic abdominal surgery: A systematic review and meta-analysis with colorectal cancer subgroup analysis. J. Robot. Surg..

[15] Elhadidi A., Shetiwy M., Al-Katary M. (2025). Comparative analysis of laparoscopic, retro-muscular, and open mesh repair techniques for ventral and incisional hernias: A comprehensive review and meta-analysis. Updates Surg..

[16] Antic A., Kmezic S., Nikolic V., Radenkovic D., Markovic V., Pejovic I., Aleksic L., Loncar Z., Antic S., Kovac J. (2022). Quality of life following two different techniques of an open ventral hernia repair for large hernias: A prospective randomized study. BMC Surg..

[17] Liang M.K., Berger R.L., Li L.T., Davila J.A., Hicks S.C., Kao L.S. (2013). Outcomes of laparoscopic vs open repair of primary ventral hernias. JAMA Surg..

[18] Sauerland S., Walgenbach M., Habermalz B., Seiler C.M., Miserez M. (2011). Laparoscopic versus open surgical techniques for ventral or incisional hernia repair. Cochrane Database Syst. Rev..

[19] Kumar A., Kuppusami B.R., Ahmad S. (2025). Evaluation of Coagulation Profile Alterations in Patients Undergoing Laparoscopic Cholecystectomy with Carbon Dioxide (CO_2_) Pneumoperitoneum. Cureus.

[20] Pochhammer J., Scholtes B., Keuler J., Müssle B., Welsch T., Schäffer M. (2020). Serum C-reactive protein level after ventral hernia repair with mesh reinforcement can predict infectious complications: A retrospective cohort study. Hernia.

[21] Fiore M., Cosenza G., Romano F.M., Pota V., Sansone P., Coppolino F., Selvaggi L., Selvaggi F., Pace M.C. (2026). Presepsin as a Novel Biomarker in Abdominal Sepsis: Diagnostic Accuracy and Prognostic Implications. Biomedicines.

[22] Di Vita G., D’Agostino P., Patti R., Arcara M., Caruso G., Davì V., Cillari E. (2005). Acute inflammatory response after inguinal and incisional hernia repair with implantation of polypropylene mesh of different size. Langenbeck’s Arch. Surg..

[23] Sijwali K., Kala S., Jauhari R.K. (2025). Comparative Study Between Extended-View Totally Extraperitoneal Rives-Stoppa Repair and Intraperitoneal Onlay Mesh Plus Repair for Ventral Abdominal Wall Hernias: A Randomized Controlled Trial. Cureus.

[24] Hassan J., Khan S., Zahra R., Razaq A., Zain A., Razaq L., Razaq M. (2022). Role of Procalcitonin and C-reactive Protein as Predictors of Sepsis and in Managing Sepsis in Postoperative Patients: A Systematic Review. Cureus.

[25] Mandal L.K., Upadhya P.S., Luitel P., Paudel S., Shah B.K., Thapaliya I., Sah S.P., Gupta R.K., Adhikary S. (2026). Quality of life following laparoscopic vs. open ventral hernia repair: A prospective comparative cohort study. J. Minimal Access Surg..

[26] Bindal V., Pandey D., Gupta S., Agarwal P., Dahiya A., Gupta D., Bindal U.D. (2025). A Real-World Experience of the Short-Term Clinical Outcomes of Laparoscopic and Robotic-Assisted Ventral Hernia Repairs. Cureus.

[27] Awaiz A., Rahman F., Hossain M.B., Yunus R.M., Khan S., Memon B., Memon M.A. (2015). Meta-analysis and systematic review of laparoscopic versus open mesh repair for elective incisional hernia. Hernia.

[28] Bittner R., Bain K., Bansal V.K., Berrevoet F., Bingener-Casey J., Chen D., Chen J., Chowbey P., Dietz U.A., de Beaux A. (2019). Update of Guidelines for laparoscopic treatment of ventral and incisional abdominal wall hernias (International Endohernia Society (IEHS))-Part A. Surg. Endosc..

[29] Othman I.H., Metwally Y.H., Bakr I.S., Amer Y.A., Gaber M.B., Elgohary S.A. (2012). Comparative study between laparoscopic and open repair of paraumbilical hernia. J. Egypt. Soc. Parasitol..

[30] Barbu L.A., Mihalache D.I., Vasile L., Mogoantă S.S., Țenea Cojan T.S., Mărgăritescu N.D., Mogoș G.F.R. (2026). Determinants of Early Quality-of-Life Improvement After Open Abdominal Wall Eventration Repair: A Large Single-Center Cohort Study Using the EuraHS-QoL Score. J. Clin. Med..

[31] de Jong D.L.C., Wegdam J.A., Berkvens E.H.M., de Vries Reilingh T.S., Nienhuijs S.W. (2025). Does quality of life improve after complex incisional hernia repair? A systematic review. Hernia.

[32] Wieland L., Alfarawan F., Bockhorn M., El-Sourani N. (2024). Comparison of eTEP and IPOM for ventral hernia surgery in the early postoperative period: A retrospective cohort study of a tertiary university centre. Hernia.

[33] Martins M.R., Santos-Sousa H., do Vale M.A., Bouça-Machado R., Barbosa E., Sousa-Pinto B. (2024). Comparison between the open and the laparoscopic approach in the primary ventral hernia repair: A systematic review and meta-analysis. Langenbeck’s Arch. Surg..

[34] Elashry M.A., Rady M., Abdelaziz A.M., Abbas M., Nasr M., Taleb H.A. (2022). Laparoscopic (IPOM and IPOM Plus) versus open para-umbilical hernia mesh repair. J. Pharm. Negat. Results.

