# Supplementary material for: Systemic Stress Biomarkers and Patient-Reported Outcomes Following Laparoscopic IPOM+ and Open Rives–Stoppa Repair for Ventral Incisional Hernia: A Prospective Non-Randomized Comparative Study
Source: J Clin Med. 2026 Sep 16;15(18):7198. doi: 10.3390/jcm15187198 (PMC13607566; doi:10.3390/jcm15187198)
Supplement: Supplementary file 1 [file jcm-15-07198-s001.zip › jcm-4433678-supplementary.pdf]

Supplementary Table S1. Adjusted operative time and postoperative outcomes according to the surgical technique.

Variable	IPOM+ (n=48) mean±SE	RS (n=48) mean±SE	Difference (95% CI)	P *adjusted*	Effect size *adjusted*
Operative time (min)	55.54±1.96	74.56±1.96	-19.021 (-24.660 to -13.381)	<0.001	0.333 (large)
Postoperative hospital stay (days)	1.86±0.19	3.56±0.19	-1.696 (-2.259 to -1.314)	<0.001	0.285 (large)
Bowel function recovery (days)	1.24±0.11	1.87±0.11	-0.629 (-0.945 to -0.314)	<0.001	0.149 (large)
Postoperative pain score (VAS, 0–10)	3.95±0.13	6.55±0.13	-2.606 (-2.989 to -2.23)	<0.001	0.670 (large)

Data are presented as mean ± standard error or mean (95% confidence interval for mean). p and effect-size measures are adjusted for age, BMI, comorbidities, employment status, and ASA. Effect size corresponds to Partial ηp^2.

Table S2. Adjusted intraoperative and postoperative complications according to surgical technique.

Complications	IPOM+ (n=48) n (p)	RS (n=48) n (p)	Difference (95% CI)	P *adjusted*	Effect size *adjusted*
Intraoperative	2 (0.48)	3 (0.56)	-0.009 (-0.101 to 0.0840)	0.854	0.000 (very small)
Postoperative	5 (0.11)	9 (0.18)	-0.125 (-0.267 to 0.017)	0.387	0.008 (very small)

Effect size corresponds to Partial ηp^2.

Table S3. Adjusted postoperative EuraHS-QoL scores according to the surgical technique.

EuraHS-QoL score	IPOM+ (n=48) mean±SD	RS (n=48) mean±SD	Difference (95% CI)	P *adjusted*	Effect size *adjusted*
Total	28.29±1.38	53.27±1.38	-24.984 (-28.955 to -21.013)	**<0.001**	0.635 (large)
Pain	8.50±0.47	16.60±0.47	-8.095 (-9.439 to -6.752)	**<0.001**	0.614 (large)
Restriction of activities	12.80±0.64	23.11±0.64	-10.309 (-12.164 to -8.455)	**<0.001**	0.575 (large)
Cosmetic	6.98±0.44	13.56±0.44	-6.579 (-7.859 to -5.300)	**<0.001**	0.537 (large)

Data are presented as mean ± standard deviation or mean (95% confidence interval for mean). Effect size corresponds to Partial ηp^2. Bold values indicate statistical significance after Bonferroni's correction.

Table S4. Nonadjusted and adjusted time to resumption to daily activities (days) according to the surgical technique.

Return to daily activities, (days)	**IPOM+ (n=48) mean±SD**	**RS (n=48) mean±SD**	**Difference (95% CI)**	**P**	**Effect size**
nonadjusted	5.17±0.95	12.21±3.00	-7.042 (-7.945 to -6.139)	**<0.001**	-3.161 (large)
adjusted	5.14±0.33	12.23±0.33	-7.088 (-8.044 to -6.131)	**<0.001**	0.707 (large)

Data are presented as mean ± standard deviation or mean (95% confidence interval for mean). Effect size corresponds to Cohen's d or Partial ηp^2. Bold values indicate statistical significance after Bonferroni's correction.

Table S5. Adjusted preoperative and postoperative biomarker levels according to surgical approach and time×group interaction effects.

**Biomarker**		**IPOM+ (n=48)**	**RS (n=48)**	**P** *adjusted*	**Effect size** *adjusted*
Cortisol (nmol/L)	preoperative	431.27±28.62	503.91±29.62	0.849	<0.001 small
	postoperative	232.36±42.46	290.84±43.94		
	change (95%CI)	198.91 (99.56 to 298.26)	213.06 (110.25 to 315.87)		
ACTH (pg/mL)	preoperative	33.34±3.47	30.22±2.94	0.333	0.011 small
	postoperative	19.54±5.25	10.52±1.70		
	change (95%CI)	13.10 (2.77 to 23.426)	20.379 (10.17 to 30.59)		
Prolactin (mIU/L)	preoperative	266.81±27.25	225.94±27.25	0.219	0.017 small
	postoperative	320.39±34.27	331.80±34.27		
	change (95%CI)	-53.58 (-111.48 to 4.313)	-105.86 (-163.76 to -47.97)		
IL-6 (pg/mL)	preoperative	5.33±0.75	7.30±0.75	0.016	0.063 moderate
	postoperative	15.17±4.41	32.49±4.41		
	change (95%CI)	-9.84 (-18.34 to -1.32)	-25.19 (-33.71 to -16.67)		
WBC (×10^9/L)	preoperative	7.67±0.31	7.12±0.31	0.454	0.006 small
	postoperative	11.57±0.50	11.46±0.50		
	change (95%CI)	-3.90 (-4.69 to -3.10)	-4.33 (-5.51 to -3.53)		
D-dimer (ng/mL)	preoperative	129.01±22.90	180.67±22.90	0.649	0.003 small
	postoperative	338.48±97.77	447.17±97.77		
	change (95%CI)	-209.47 (-380.49 to -38.45)	-266.501 (-437.52 to -95.48)		
Glucose (mmol/L)	preoperative	6.32±0.28	6.01±0.28	0.177	0.020 small
	postoperative	5.42±0.20	5.66±0.20		
	change (95%CI)	0.90 (0.35 to 1.45)	0.35 (0.20 to 0.91)		
CRP (mg/L)	preoperative	4.26±0.97	5.32±0.98	0.043	0.045 moderate
	postoperative	22.36±4.97	37.17±5.02		
	change	-18.10	-31.85		

	(95%CI)	(-27.21 to -8.98)	(-41.06 to -22.63)		
Presepsin (pg/mL)	preoperative	219.92±20.09	254.42±20.32	0.041	0.046 small
	postoperative	356.70±44.77	517.75±45.27		
	change (95%CI)	-136.78 (-220.11 to -53.45)	-263.33 (-347.58 to -179.07)		
Procalcitonin (ng/mL)	preoperative	0.034±0.004	0.047±0.004	0.050	0.042 small
	postoperative	0.086±0.021	0.158±0.020		
	change (95%CI)	-0.05 (-0.09 to -0.01)	-0.11 (-0.15 to -0.07)		

Data are presented as mean ± standard error or mean (95% confidence interval for mean). Effect size corresponds to Partial ηp^2. The p values and partial eta squared (ηp^2) values refer to the time×surgical approach interaction obtained using repeated-measures ANOVA. Bold values indicate statistical significance after Bonferroni's correction.
